# Dyspepsia in nonagenarian women

**DOI:** 10.1007/s41999-025-01197-w

**Published:** 2025-04-10

**Authors:** Ersin Kuloglu, Ilker Sengul, Demet Sengul, Ali Muhtaroglu, Sefer Aslan, Kubilay Issever, Ahmet Cumhur Dulger

**Affiliations:** 1 Department of Internal Medicine, Faculty of Medicine, Giresun University, Gazipasa Compound, Gazi Avenue, 28100 Giresun, Turkey; 2 Division of Endocrine Surgery, Faculty of Medicine, Giresun University, Giresun, Turkey; 3 Department of General Surgery, Faculty of Medicine, Giresun University, Giresun, Turkey; 4 Department of Pathology, Faculty of Medicine, Giresun University, Giresun, Turkey; 5 Division of Gastroenterology, Faculty of Medicine, Giresun University, Giresun, Turkey

**Keywords:** Dyspepsia, Nonagenarians, *Helicobacter pylori*, Pathology, Geriatrics

## Abstract

**Purpose:**

Dyspeptic complaints are common across all age groups, but limited research explores their characteristics and underlying pathologies in nonagenarians. This pioneering study aims to investigate the clinical, laboratory, and histopathological features in women nonagenarians vs. middle-aged.

**Methods:**

A cross-sectional study included 93 female nonagenarians and 90 under 65 years (control), all presenting with dyspeptic complaints to general surgery, internal medicine, and gastroenterology outpatient clinics. Both groups underwent comprehensive evaluations, including laboratory parameters, upper gastric endoscopic examinations, and histopathological assessments of gastric biopsies. Statistical analysis compared the two groups' demographic, laboratory, and histopathological findings.

**Results:**

Of the patients included in the study, 49.8% were under 65 years, and 50.2% were nonagenarians. The mean age of the control group was 54.8 years, while nonagenarians had a mean age of 92.7 years. Significant differences were observed in several laboratory biomarkers such as leukocytes, neutrophils, glucose, creatinine, sodium, potassium, albumin, alanine transaminase, C-reactive protein, etc. between the groups (*p* < 0.05). Nonagenarians were less infected with *Helicobacter pylori*, whereas they had a higher frequency of intestinal metaplasia.

**Conclusions:**

Nonagenarians group has a higher rate of intestinal metaplasia and displasia, while a lower rate of *Helicobacter pylori* infection in their gastric mucosal specimens. More extensive randomized controlled trials should illuminate the possible pathophysiological mechanisms for this association.

**Supplementary Information:**

The online version contains supplementary material available at 10.1007/s41999-025-01197-w.

## Key summary points

**Aim:**

This study aims to investigate the clinical, laboratory, and histopathological features in women nonagenarians vs. middle-aged.

**Findings:**

Significant differences were observed in several laboratory biomarkers such as leukocytes, neutrophils, glucose, creatinine, sodium, potassium, albumin, alanine transaminase, C-reactive protein, etc. between the groups (*p* < 0.05). Nonagenarians were less infected with Helicobacter pylori, whereas they had a higher frequency of intestinal metaplasia.

**Message:**

Nonagenarians group has a higher rate of intestinal metaplasia and displasia, while a lower rate of Helicobacter pylori infection in their gastric mucosal specimens. More extensive randomized controlled trials should illuminate the possible pathophysiological mechanisms for this association.

**Supplementary Information:**

The online version contains supplementary material available at 10.1007/s41999-025-01197-w.

## Introduction

Dyspepsia, *per* se, characterized by chronic or recurrent pain in the upper abdomen, bloating, and nausea, is a common clinical complaint affecting millions worldwide, irrespective of age [1]. While the prevalence and pathophysiology of dyspepsia have been extensively studied in the general population, there still needs to be a significant gap in our understanding of its impact on the nonagenarian population, individuals who are 90 years of age or older. This population is rapidly growing, yet their unique healthcare needs, particularly concerning gastroenterological disorders, need to be more well-documented and understood.

Dyspepsia's clinical manifestation is diverse, ranging from epigastric pain and discomfort to bloating, early satiety, and nausea, impacting the patient's quality of life significantly [2]. As such, its incidence increases with age, affecting approximately 25–40% of the older population, reflecting both the complexity and the increased vulnerability of this age group to digestive disorders [3]. The etiology of dyspepsia in the older people is multifactorial, encompassing both organic causes, such as peptic ulcer disease, gastroesophageal reflux disease (GERD), gastric cancer, and functional dyspepsia, where no apparent organic cause can be identified. Pathophysiologically, aging is associated with several gastrointestinal changes, including reduced gastric acid secretion, impaired gastric and intestinal motility, and increased prevalence of *Helicobacter pylori* [*H. pylori*] infection, contributing to dyspeptic symptoms [4]. Moreover, the impact of polypharmacy, a common issue in geriatric care, cannot be understated, as many medications used by the older people have gastrointestinal side effects that can exacerbate or mimic dyspeptic symptoms [5]. Despite its prevalence and impact, the diagnosis and management of dyspepsia in the older people remain challenging, often requiring a nuanced approach to accommodate the unique physiological and psychological aspects of aging. This study represents a comprehensive and comparative analysis of dyspepsia in nonagenarian women vs. middle-aged group.

## Methods

### Ethical aspects

The study protocol was approved by the local ethics committee linked to the Giresun Education and Research Hospital under the 243290198.08.05.2024/06 approval number. Due to the retrospective nature of the study, informed consent was waived. This study adhered to the ethical principles outlined in the 2013 revision of the Declaration of Helsinki. It was conducted at Giresun Education and Research Hospital in Turkey.

### Study design

Our retrospective analysis incorporated 183 females admitted to our outpatient clinics of Internal Medicine, General Surgery, and Gastroenterology with dyspeptic complaints between January 2020 and December 2022. Among these patients, 93 women aged 90 years and above were classified as nonagenarians, while the remaining 90 women under 65 years were assigned to the control group. In the nonagenarian group applying to our hospital, male patients were very few. We decided to exclude a few male patients in this study to eliminate the confounding factor of gender. These 93 patients were the ones whose data we were able to access retrospectively in the nonagenarian group. The control group data were randomly selected from the available data which contains dyspeptic patients applied to the outpatient clinics between the abovementioned dates.

Patients’ demographic data, including age and sex, were collected from the medical records. Laboratory data included leukocyte count (WBC), neutrophils, lymphocytes, monocytes, hemoglobin (Hgb), mean corpuscular volume (MCV), platelet count (Plt), mean platelet volume (MPV), platelet distribution width (PDW), red blood cell distribution width (RDW), glucose, urea, creatinine, sodium (Na), potassium (K), calcium (Ca), albumin (Alb), alanine aminotransferase (ALT), aspartate aminotransferase (AST), C-reactive protein (CRP), erythrocyte sedimentation rate (ESR), thyroid stimulating hormone (TSH) and free T4 (fT4). The lab tests were performed at the time of the endoscopic procedure, and biopsy samples were obtained from all the cases during endoscopy, immediately fixed in 10% formalin, and sent to the Department of Pathology for histopathological analysis. Finally, *H. pylori*, atrophy, dysplasia, and metaplasia were evaluated and documented by experienced pathologists blinded to clinical data.

### Statistical analysis

The Statistical Package Social Sciences (SPSS) 26.0 program was used for the analysis of this study. The suitability of the numerical variables of the patients for normal distribution was decided by looking at the skewness and kurtosis values. The rules recognized normal distribution except for hemoglobin, PDW, RDW, glucose, urea, creatine, ALT, AST, and TSH values. The reference value in a normal distribution is between ± 1.96. A chi-square test was used to compare positivity of gastric biopsy results among different age groups. An independent sample *t* test or Mann–Whitney *U* test was used to compare laboratory parameters for various age groups.

Furthermore, the Pearson or Spearman Correlation tests examined the relationships between different age groups, laboratory data, and gastric biopsy results. Moreover, the correlation coefficient was evaluated as a low-level relationship between 0.00 and 0.30, a medium-level relationship between 0.30 and 0.70, and a high-level relationship between 0.70 and 1.00. In the study, significance levels were carried out by considering 0.05 and 0.01 values.

## Results

Of the patients included in the study, 49.8% were under 65 years, and 50.2% were nonagenarians. The mean age of the control was 54.8 years, while nonagenarians had a mean age of 92.7 years (*p* = 0.000). There was no significant difference in the MCV, Plt, MPV, PDW, AST, ALT, Creatinine, and TSH values of the patients between the groups (*p* > 0.05) (Table 1). According to these results, the mean and median values of WBC, neutrophil, monocyte, RDW, glucose, urea, CRP, ESR, and fT4 values of nonagenarians and lymphocyte, Hgb, Na, K, Ca, and Alb values of the control were significantly higher The control group had a significantly higher rate of H.pylori positivity but a considerably lower rate of intestinal metaplasia positivity compared to the older people. No significant differences were observed in atrophy and dysplasia rates, which were higher in nonagenarians.

**Table 1:** Comparison of gastric biopsy and laboratory results between the control and nonagenarians

Variables		Control (*n*: 90)		Nonagenarians (*n*: 93)		*p*
		Number	%	Number	%	
*H. pylori*	Negative	57	63.3	82	88.2	0.000**
	Positive	33	36.7	11	11.8	
Intestinal metaplasia	Negative	84	93.3	74	79.6	0.013*
	Positive	6	6.7	19	20.4	
Atrophy	Negative	82	91.1	82	88.2	0.682
	Positive	8	8.9	11	11.8	
Dysplasia	Negative	90	100.0	88	94.6	0.059
	Positive	0	0.0	5	5.4	
Hgb^t^ g/dL		12.6 ± 1.6		10.4 ± 1.8		0.000**
CRPt mg/L		3.7 ± 5.0		42.8 ± 43.4		0.000**
ESRt mm/hour		25.2 ± 16.1		45.6 ± 24.1		0.000**

The *p*-value of the parameters with statistically significant difference is shown in bold

*p < 0,05, **p < 0,01, *χ*^*2*^*:* Chi-square test (categorical data)

## Discussion

The findings from our comparative analysis provide valuable insights into the characteristics of dyspepsia in nonagenarians compared to middle-aged groups, revealing both expected and unexpected patterns. Notably, the decreased prevalence of *H. pylori* positivity in nonagenarians (17.1%) compared to the control [38.5%] stands in contrast to anticipated outcomes based on existing literatüre [6, 7]. Traditionally, the prevalence of *H. pylori* infection is thought to increase with age, attributed to less effective immune responses and the cumulative likelihood of acquiring the infection over a longer lifetime. However, our findings echo a study by Malfertheiner et al., which suggested a decline in *H. pylori* prevalence among the oldest population, possibly due to a cohort effect or increased mortality among infected individuals. Conversely, the higher rates of intestinal metaplasia and dysplasia observed in the older people could reflect a prolonged exposure to risk factors not directly linked to *H. pylori* presence, such as dietary habits, environmental factors, or the natural decline in gastric mucosal defense mechanisms with age. These findings align with the work of Correa, who proposed a progression model for gastric carcinogenesis starting from chronic gastritis to intestinal metaplasia, dysplasia, and cancer, highlighting the significance of our observations in understanding gastric cancer risk in the older people [8–15].

Our results also demonstrated significant differences in various laboratory parameters between the two age groups. When comparing laboratory parameters between the two groups, we based our analysis on two hypotheses. The first was to determine whether the differences in gastric mucosa between the two groups could be explained by the laboratory parameters at the time of admission from a pathophysiological perspective. The second was to understand whether there is any relationship or correlation between gastric mucosa changes and laboratory parameters. This may reflect the complex interplay of physiological aging, chronic diseases, and differential responses to stress and infections [2]. With aging, the prevalence of anemia increases due to potential malnutrition, iron deficiency, chronic diseases, and occult bleeding from gastrointestinal mucosal lesions [16]. In this study, nonagenarians had significantly higher frequency of anemia. The increase in markers such as leukocytes, neutrophils, monocytes, CRP, and ESR in the older group underscores the heightened inflammatory state, known as "inflammaging," a term coined by Franceschi et al. to describe the low-grade, chronic, systemic inflammation characterizing aging [17]. The higher prevalence of chronic diseases, worse nutritional status, and increased frequency of dehydration in nonagenarians may explain the worse results of acute-phase reactants such as CRP and ESR in this group. The higher frequency of intestinal metaplasia and dysplasia in this group may be related to the associated high inflammation. This state may contribute to the pathophysiology of dyspepsia and its complications in the older people [18].

Furthermore, as recent studies have suggested, the observed alterations in hematological and biochemical markers across age groups emphasize the need for age-specific reference ranges in clinical practice [19]. Such tailored diagnostic criteria are crucial for accurately interpreting test results in older patients and improving the management of dyspeptic complaints and associated conditions [20–22].

.Interestingly, the lack of significant correlation between specific parameters (MCV, MPV, PDW, AST, TSH) and advanced age might indicate that these factors are less influenced by aging or the pathophysiological processes underpinning dyspepsia in the older people. This observation aligns with previous research suggesting that these parameters are stable across different age spectrums [23–25].

In conclusion, this study not only sheds light on the unique clinical and laboratory profiles of dyspepsia in nonagenarians but also challenges some preconceived notions about the prevalence of *H. pylori* infection in the older people. The increased rates of intestinal metaplasia and dysplasia in this age group underscore the importance of vigilant screening and monitoring strategies. Future research should emphasize longitudinal studies to investigate the dynamics of gastric pathologies in aging populations and to develop tailored treatment strategies that address the unique needs of the older people.

As we explore geriatric gastroenterology further, our findings underscore the necessity of integrating clinical insights with a broader understanding of aging biology. The journey toward optimizing care for our oldest patients is challenging and rewarding, offering new medical research and practice horizons.

While pioneering in its comparative analysis of dyspepsia in nonagenarians vs. younger age groups, this study has limitations. The first is including only female patients in the nonagenarian group. Due to the significantly higher proportion of women and the very limited number of men in nonagenarians in our region, this study was designed to include only female patients. For this reason, only female patients were included to prevent gender from being a confounding or heterogeneous factor that could disrupt the objectivity of the results. Therefore, including only female patients in the study group limits the generalizability of the results. The second is the lack of standardization of laboratory parameters according to age groups. The cross-sectional design limits our ability to infer causality or track the progression of dyspeptic conditions over time. In addition, the study's reliance on patients from outpatient clinics may not fully represent the broader older population, particularly those with limited access to healthcare services or residing in long-term care facilities**.** Finally, the absence of geriatric assessment tools in our outpatient clinics and the retrospective nature of this study prevented the evaluation of geriatric factors, such as nutritional status and cognitive level. Considering these limitations, further studies with larger patient groups are needed to generalize the interesting results obtained in this rare-to-find, specialized group.

## Conclusions

Our findings of this unique study revealed that dyspeptic nonagenarian patients had a lower incidence of *H. pylori* infection than the dyspeptic middle-aged. This unexpected result should be confirmed, and future studies involving larger patient populations should highlight the potential underlying associations. According to upcoming research in this area, the management of dyspepsia in the geriatric population should be reassessed.

## Supplementary Information

Below is the link to the electronic supplementary material.

Supplementary file1 (DOCX 17 KB)

## Data Availability

The data sets generated and/or analysed during the current study are not publicly available. However, the data sets are available from the corresponding author on reasonable request.
