# Supplementary material for: Dyspepsia in nonagenarian women
Source: Eur Geriatr Med. 2025 Apr 10;16(5):1811–6. doi: 10.1007/s41999-025-01197-w (PMC12528204; doi:10.1007/s41999-025-01197-w)
Supplement: Supplementary file 1 — Supplementary file1 (DOCX 17 KB) [file 41999_2025_1197_MOESM1_ESM.docx]

The comparison of laboratory parameters between control and nonagenarians

| *Laboratory parameters* | *Control (n: 90)* | *Nonagenarians*  *(n: 93)* | *p* |
| --- | --- | --- | --- |
|  | *Med..± S.D.* | *Med..± S.D.* |  |
| WBC^t^ /mm^3^ | 7,2 ± 1,9 | 9,2 ± 4,2 | **0,000**** |
| Neutrophils^z^ /mm^3^ | 4,2 ± 1,6 | 7,0 ± 4,4 | **0,000**** |
| Lymphocytes^t^ /mm^3^ | 2,2 ± 0,7 | 1,5 ± 0,8 | **0,000**** |
| Monocytes^t^ /mm^3^ | 0,3 ± 0,1 | 0,5 ± 0,2 | **0,000**** |
| MCV^t^ fL | 84,9 ± 7,5 | 87,0 ± 7,2 | 0,056 |
| Plt^t^ /mm^3^ | 282,6 ± 74,0 | 263,4 ± 91,2 | 0,121 |
| MPV^t^ fL | 9,4 ± 1,0 | 9,3 ± 1,2 | 0,817 |
| PDW^z^ % | 15,3 ± 2,1 | 15,6 ± 1,4 | 0,611 |
| RDW^z^ % | 14,1 ± 1,9 | 16,2 ± 3,3 | **0,005**** |
| Glucose^z^ mg/dL | 106,6 ± 21,5 | 121,6 ± 36,8 | **0,001**** |
| Urea^z^ mg/dL | 29,2 ± 10,5 | 57,3 ± 35,4 | **0,000**** |
| Creatinine^z^ mg/dL | 0,6 ± 0,1 | 0,7 ± 0,3 | 0,170 |
| Na^z^ mmol/L | 140,8 ± 2,0 | 138,1 ± 5,3 | **0,000**** |
| K^t^ mmol/L | 4,4 ± 0,4 | 4,0 ± 0,6 | **0,000**** |
| Ca^t^ mg/dL | 9,6 ± 0,5 | 8,6 ± 0,8 | **0,000**** |
| Alb g/L | 44,3 ± 3,8 | 31,5 ± 6,2 | **0,000**** |
| ALT^z^ U/L | 19,7 ± 10,2 | 19,3 ± 16,8 | 0,821 |
| AST^z^ U/L | 20,5 ± 8,6 | 23,8 ± 15 | 0,068 |
| TSH^z^ mu/L | 1,6 ± 1,3 | 1,4 ± 1,2 | 0,212 |
| FT4^t^ ng/dL | 1,1 ± 0,2 | 1,2 ± 0,2 | **0,012*** |

*p<0,05, **p<0,01, t: Independent Sample T-Test, z: Mann Whitney U Test
